# Image-based mandibular and maxillary parcellation and annotation using computed tomography (IMPACT): a deep learning-based clinical tool for orodental dose estimation and osteoradionecrosis assessment

**DOI:** 10.1016/j.phro.2025.100817

**Published:** 2025-07-25

**Authors:** Laia Humbert-Vidan, Austin H. Castelo, Renjie He, Lisanne V. van Dijk, Dong Joo Rhee, Congjun Wang, He C. Wang, Kareem A. Wahid, Sonali Joshi, Parshan Gerafian, Natalie West, Zaphanlene Kaffey, Sarah Mirbahaeddin, Jaqueline Curiel, Samrina Acharya, Amal Shekha, Praise Oderinde, Alaa M.S. Ali, Andrew Hope, Erin Watson, Ruth Wesson-Aponte, Steven J. Frank, Carly E.A. Barbon, Kristy K. Brock, Mark S. Chambers, Muhammad Walji, Katherine A. Hutcheson, Stephen Y. Lai, Clifton D. Fuller, Mohamed A. Naser, Amy C. Moreno, for the OPC-SURVIVOR Program, Renjie He, Steven J. Frank, Carly E.A. Barbon, Kristy K. Brock, Mark S. Chambers, Katherine A. Hutcheson, Stephen Y. Lai, Clifton D. Fuller, Mohamed A. Naser, Amy C. Moreno, the MD Anderson Head and Neck Cancer Symptom Working Group, Laia Humbert-Vidan, Renjie He, Kareem A. Wahid, Natalie West, Zaphanlene Kaffey, Alaa M.S. Ali, Ruth Wesson-Aponte, Carly E.A. Barbon, Mark S. Chambers, Katherine A. Hutcheson, Stephen Y. Lai, Clifton D. Fuller, Mohamed A. Naser, Amy C. Moreno

**Affiliations:** i Division of Radiation Oncology, The University of Texas MD Anderson Cancer Center, Houston, TX, USA; k Department of Head and Neck Surgery, The University of Texas MD Anderson Cancer Center, Houston, TX, USA; j Department of Imaging Physics, The University of Texas MD Anderson Cancer Center, Houston, TX, USA; l Division of Radiation Oncology, The University of Texas MD Anderson Cancer Center, Houston, TX, USA; m Department of Imaging Physics, The University of Texas MD Anderson Cancer Center, Houston, TX, USA; n Department of Head and Neck Surgery, The University of Texas MD Anderson Cancer Center, Houston, TX, USA; a Division of Radiation Oncology, The University of Texas MD Anderson Cancer Center, Houston, TX, USA; b Department of Imaging Physics, The University of Texas MD Anderson Cancer Center, Houston, TX, USA; c Department of Radiation Oncology, University Medical Center Groningen, Groningen, Netherlands; d Department of Radiation Physics, The University of Texas MD Anderson Cancer Center, Houston, TX, USA; e Department of Radiation Oncology, Princess Margaret Cancer Center, Toronto, CA, USA; f Department of Dental Oncology, Princess Margaret Cancer Center, Toronto, CA, USA; g Department of Head and Neck Surgery, The University of Texas MD Anderson Cancer Center, Houston, TX, USA; h Department of Clinical and Health Informatics, Texas Center of Oral Health Care Quality & Safety, Houston, TX, USA

**Keywords:** Head and neck cancer, Radiotherapy, Deep-learning auto-segmentation models, Orodental structures, Osteoradionecrosis

## Abstract

**Background and purpose:**

Accurate delineation of orodental structures on radiotherapy computed tomography (CT) images is essential for dosimetric assessment and dental decisions. We propose a deep-learning (DL) auto-segmentation framework for individual teeth and mandible/maxilla sub-volumes aligned with the ClinRad osteoradionecrosis staging system.

**Materials and methods:**

Mandible and maxilla sub-volumes were manually defined on simulation CT images from 60 clinical cases, differentiating alveolar from basal regions; teeth were labelled individually. For each task, a DL segmentation model was independently trained. A Swin UNETR-based model was used for mandible sub-volumes. For smaller structures (e.g., teeth and maxilla sub-volumes) a two-stage model first used the ResUNet to segment the entire teeth and maxilla regions as a single ROI used to crop the image input for Swin UNETR. In addition to segmentation accuracy and geometric precision, a dose-volume comparison was made between manual and model-predicted segmentations.

**Results:**

Segmentation performance varied across sub-volumes – mean Dice values of 0.85 (mandible basal), 0.82 (mandible alveolar), 0.78 (maxilla alveolar), 0.80 (upper central teeth), 0.69 (upper premolars), 0.76 (upper molars), 0.76 (lower central teeth), 0.70 (lower premolars), 0.71 (lower molars) – with limited applicability in segmenting sub-volumes absent in the data. The maxilla alveolar central sub-volume showed a statistically significant dose-volume difference in both D_mean_ and D_2%_.

**Conclusions:**

We present a novel DL-based auto-segmentation framework of orodental structures, enabling spatial localization of dose-related differences. This tool may enhance image-based bone injury detection and improve clinical decision-making in radiation oncology and dental care for head and neck cancer patients.

## 1 Introduction

The introduction of modern imaging and radiation therapy techniques has enabled highly conformal irradiation of head and neck tumors but, due to the close proximity of critical structures in that region, non-target organs such as the mandible or maxilla inevitably absorb ionizing radiation, resulting in detrimental complications such as osteoradionecrosis (ORN). Radiation-induced injury to the mandible and maxilla results from direct cellular and vascular damage to the bone, resulting in reduced blood supply and subsequent necrosis of the bone tissue (i.e., ORN). Thus, the healing process occurring after a dental extraction or any physical trauma to the bone may be slower or absent in irradiated bone [1]. Such considerations are important in dental (e.g., dental extractions or implants) and oncological (e.g., radiation dose distribution) decisions made pre-, during, and post-treatment of patients with head and neck cancer [[2], [3], [4]].

Accurate delineation of orodental structures on radiotherapy computed tomography (CT) images is essential for effective dose-volume evaluation during treatment planning optimization and also post-treatment to support dental decisions, such as evaluating the feasibility of dental implants or assessing the risk of normal tissue damage following irradiation. Manual segmentation of orodental structures is a time-consuming, poorly reproducible and often challenging task due to strong artifacts caused by high density (higher attenuation) materials such as dental fillings, restorations or titanium implants, which result in blurred edges of the structure[5]. Boundaries between adjacent teeth are often indistinct, the angles of teeth may diverge from crown to root, and positional variations, such as gaps or missing teeth, can further complicate the process. This has led to increased research focus in recent years of developing automated segmentation methods for orodental structures, mostly mandible [[5], [6], [7]] and teeth [[8], [9], [10], [11], [12], [13]].

These studies, however, considered the mandible as a single structure disregarding its heterogeneity with regards to composition and radiobiological characteristics. The bone composition varies across the mandible and maxilla, with density, metabolic and radiosensitivity differences between sub-volumes. For instance, the alveolar process surrounding the teeth has a higher proportion of spongy or trabecular bone, which is more vascularized and metabolically active and is more sensitive to radiation as it contains bone marrow [14]. The basal region has a denser composition, with a larger proportion of cortical bone [15].

On the other hand, most teeth auto-segmentation studies leverage cone-beam CT data, which is considered the gold standard for volumetric dental imaging. Limited series have focused on teeth auto-segmentation specifically to support radiation oncology for head and neck cancer patients utilizing surveillance contrast-enhanced CT images and/or radiation simulation CT, which pose additional challenges due to lower spatial resolution in bone compared to cone-beam CT yet are more clinically relevant for this patient population.

In this study, we present a novel and comprehensive approach to the auto-segmentation of bony odontic structures on CT images, aimed at supporting clinical decision-making in radiation therapy for patients with head and neck cancer. Advancing beyond existing whole mandible auto-segmentation methods [[5], [6], [7], [8]], we propose a refined definition of mandible and maxilla sub-volumes that accounts for variations in bone composition and the anatomical and physiological differences within these structures. Our proposed sub-volumes are designed to align with the recently ASCO-endorsed ORN staging system, the ClinRad system [16], which incorporates radiological assessment of the vertical extent of bone damage. This alignment has the potential to improve early detection of mandible and maxilla damage, particularly in cases with intact mucosa, providing a more clinically meaningful segmentation framework.

## 2 Materials and methods

### 2.1 Patients

After institutional review board approval (RCR030800), data for 60 cases from a philanthropically funded observational cohort (Stiefel Oropharynx Cancer Cohort, PA14-0947) were extracted retrospectively. Cases with more than 20 missing teeth or with overt image artifacts (i.e., streak artifact preventing from accurate manual teeth delineation) were excluded. Radiotherapy treatment data included planning CT images, structure sets and planned radiation dose distributions. Clinical treatment plans had been created using the RayStation (RaySearch Laboratories, Sweden), Eclipse (Varian Medical Systems, Palo Alto, CA, USA) and Pinnacle (Philips Radiation Oncology Systems, Fitchburg, WI, USA) treatment planning systems (TPS).

### 2.2 Contouring

The contouring methodology was agreed amongst a group of clinical experts (LHV, ACM, CDF, EW, AH, RWA). Structures were manually contoured by 10 observers of varying degree of expertise, including undergraduate (SJ, PG) and postgraduate (SM, JC, SA, AS, PO) students and doctoral (NW, ZK) and postdoctoral (AMSA) trainees, following detailed contouring instructions (see Supplement A) and an associated RayStation structure set template for consistency. All contours were qualitatively reviewed by two clinical experts (LHV, ACM).

#### 2.2.1 Mandible and maxilla sub-volumes

**Fig. 1:**
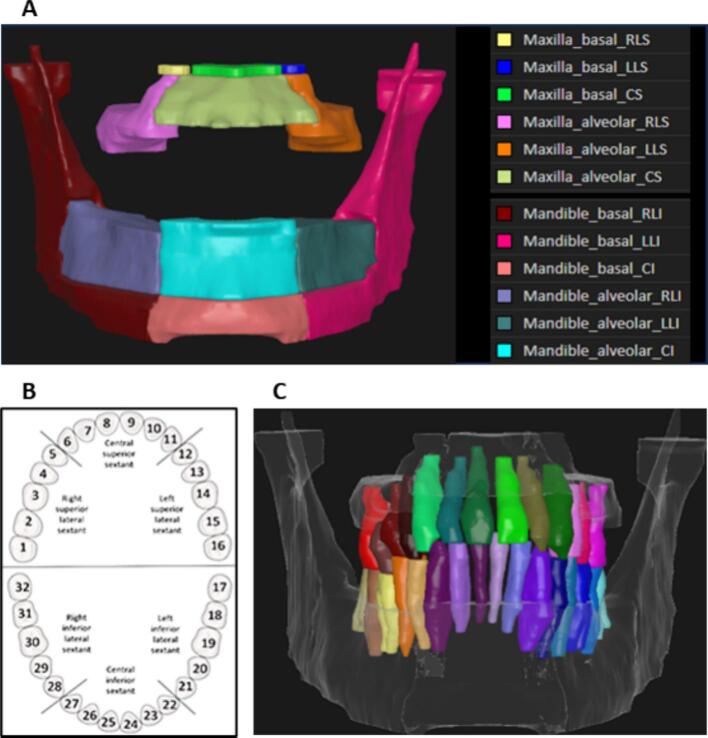
Orodental structures definitions utilized. The illustrations show A) maxilla and mandible sub-volumes defining left/right/central alveolar and basal bone regions, where laterality of the sub-volumes was defined by grouping the teeth into three sextants (B), and C) individual teeth segmentation including the roots extending into the mandible and maxilla bones, where teeth were numbered following the Universal Numbering System (1–32).

Mandible and maxilla sub-volumes were manually contoured on planning CT images in RayStation TPS to include left/right/central alveolar and basal bone regions, i.e., a total of 12 sub-volumes were obtained per case; laterality of the sub-volumes was defined by grouping the teeth into three sextants (Fig. 1). A more detailed description of the sub-volume contouring process is provided in Supplements A and B. The alveolar region was initially defined as an expansion from the alveolar crest of 5 mm inferiorly and 3 mm superiorly for the mandible and maxilla, respectively [15]. However, at the level of the mandibular molars, the alveolar ridge is typically shorter, and applying a 5 mm inferior expansion often led to overlap with the basal bone. To address this, the contours of the lateral alveolar sub-volumes were manually adjusted for each patient by reducing the expansion from 5 mm to 4 mm (see Supplementary Fig. S3). This adjustment ensured accurate differentiation between alveolar and basal regions, thus aligning with the ClinRad ORN classification system, and enabled continuous delineation of the basal region from the chin to the mandibular condyles via the angle.

#### 2.2.2 Teeth

Individual teeth were manually contoured and numbered following the American Dental Association (ADA) Universal Numbering System (1–32) (Fig. 1). Missing teeth were not contoured but accounted for with regards to the numbering; a tooth was considered as missing if the gap between adjacent teeth was larger than 5 mm. To ensure generalizability of the model on edentulous or semi-edentulous patients, empty tooth sockets in cases with missing teeth were included in the alveolar region by manually adjusting the teeth expansion contour (e.g., Supplementary Fig. S4).

### 2.3 Image preprocessing

DICOM (Digital Imaging and Communications in Medicine) files (CT images and RT structure set) were exported from the radiotherapy treatment planning system and converted into NIfTI (Neuroimaging Informatics Technology Initiative) format using *SimpleITK* [17] and *dcmrtstruct2nii* software [18]. Technical details on the CT scans are available in Supplement C. Structures were converted into masks and assigned individual labels; a priority-based approach (with central volumes having priority over lateral ones) was used for mandible and maxilla sub-volumes to address potential voxel overlap during the manual contouring process. All images were resampled to a fixed resolution of 1 mm in the axial plane (x, y dimensions) and 2 mm in the z dimension, with CT Hounsfield Units (HU) clipped between −200 and 1600 to enhance bone structures and rescaled between 0 and 1.

### 2.4 Model implementation

The anatomy of the different orodental structures considered varies significantly (e.g., individual teeth are significantly smaller than mandible or maxilla sub-volumes and the maxilla basal region is also smaller to that of the mandible), which led to the decision of addressing the following three segmentation tasks separately: mandible sub-volumes, maxilla sub-volumes and individual teeth. For each task, a deep-learning (DL) segmentation model was independently trained. We used the Residual U-Net (ResUNet) [19] and Swin UNETR [20] Transformer-based convolutional neural networks from the Medical Open Network for Artificial Intelligence (MONAI) software package [21]. A one-stage Swin UNETR-based model was used for the mandible sub-volumes. For the smaller structures, such as teeth and maxilla sub-volumes, a two-stage segmentation model was adopted to enhance segmentation performance and address challenges posed by class imbalance [22] and the small size of target structures. In the first stage, a ResUNet model was applied to the full, uncropped CT image to segment a coarse region of interest (ROI). This provided an estimate of the center of mass, which was then used to crop the image to a fixed voxel size of 128 × 128 × 128, thus minimizing background noise and irrelevant anatomical features, and helping the model to focus on the relevant region. In the second stage, the cropped image was input into a Swin UNETR model to perform fine-grained segmentation of individual teeth and maxilla sub-volumes.

Data augmentation included random rotations (±12°), scaling (±10 %), and random intensity scaling and shifting (±10 %). Both the one-stage and two-stage models were trained for 500 epochs with a learning rate of 10^-4^ using the Dice-Cross Entropy loss function from MONAI. Patients were randomly split into train and test subsets. A 5-fold cross-validation approach was employed, with with five sub-models applied for each task (mandible, maxilla, and teeth), and the final segmentation was obtained using a majority vote across the outputs of these models.

### 2.5 Model performance

Model performance was evaluated on the test subset with regards to segmentation accuracy and geometric precision using the Dice Similarity Coefficient (Dice), Mean Intersection over Union (mIoU), 95th percentile Hausdorff Distance (HD95) and Average Surface Distance (ASD) metrics. Dice and mIoU provide a measure of overlap between predicted segmentations and ground truth, ranging from 0 (no overlap) to 1 (perfect overlap). HD95 and ASD capture segmentation boundary precision, with smaller distances corresponding to better alignment between the predicted and ground truth surfaces, with HD95 specifically capturing the 95th percentile of surface distances to reduce sensitivity to outliers. These metrics were calculated in Python using the packages *medpy.metric.binary*, *scipy.spatial.distance*, *surface_distance.metrics* and *NumPy*.

### 2.6 Dose-volume comparison

The performance of the segmentation models was further assessed by comparing the values for the mean dose (D_mean_) and the dose received by the hottest 2 % of the volume (D_2%_) within each segmented class for both manual (i.e., ground truth) and model-predicted segmentations. Radiation dose distribution and corresponding structures files were exported from the TPS in DICOM format and converted into NIfTI format for compatibility with Python-based processing. All images (including CT, dose, and structure masks) were resampled to a common voxel resolution to ensure spatial alignment. The resampled segmentation volumes were then used to extract corresponding regions from the 3D dose distribution using voxel-wise masking operations. For each anatomical sub-region, dose metrics were computed including the mean dose (D_mean_) and the dose received by the hottest 2 % of the volume (D_2%_) using NumPy-based array operations. Comparisons between model-predicted and manually contoured structures were statistically evaluated using the Wilcoxon signed-rank test, with Bonferroni correction applied to adjust for multiple testing.

## 3 Results

### 3.1 Patients

The final cohort consisted of 60 patients treated with head and neck cancer who underwent radiotherapy with prescribed doses from 60 Gy in 30 fractions to 70 Gy in 35 fractions. Table 1 summarizes the demographic and dental characteristics of this cohort.

**Table 1:** Cohort demographic and dental characteristics for the train and test subsets.

Variable		Train dataset (N = 50)	Test dataset (N = 10)
Sex			
	Male (N, %)	47 (94 %)	5 (50 %)
	Female (N, %)	3 (6 %)	5 (50 %)
Age (median, IQR)		58.8 (53.6–63.4)	63.9 (52.6–71.2)
Smoking status			
	Never (N, %)	21 (42 %)	4 (40 %)
	Former (N, %)	28 (56 %)	6 (60 %)
	Current (N, %)	1 (2 %)	0 (0 %)
	Unknown (N, %)	0 (0 %)	0 (0 %)
Tobacco pack years (median, IQR)		26.2 (12.9–43.8)	40.0 (10.0–60.0)
Chew tobacco			
	Yes (N, %)	8 (16 %)	0 (0 %)
	No (N, %)	38 (76 %)	8 (80 %)
	Unknown (N, %)	4 (8 %)	2 (20 %)
Alcohol status			
	Yes (N, %)	36 (72 %)	7 (70 %)
		14 (28 %)	2 (20 %)
	Unknown (N, %)	0 (0 %)	1 (10 %)
Gingival recession			
	Yes (N, %)	19 (38 %)	2 (20 %)
	No (N, %)	13 (26 %)	4 (40 %)
	Unknown (N, %)	18 (36 %)	4 (40 %)
Tooth wear			
	Yes (N, %)	21 (42 %)	1 (10 %)
	No (N, %)	6 (12 %)	3 (30 %)
	Unknown (N, %)	23 (46 %)	6 (60 %)
Oral hygiene*			
	Good (N, %)	31 (62 %)	8 (80 %)
	Fair (N, %)	13 (26 %)	1 (10 %)
	Poor (N, %)	3 (6 %)	0 (0 %)
	Not reported (N, %)	3 (6 %)	1 (10 %)
Pre-RT periodontal disease			
	Yes (N, %)	18 (36 %)	1 (10 %)
	No (N, %)	22 (44 %)	5 (50 %)
	Unknown (N, %)	10 (20 %)	4 (40 %)
Pre-RT caries			
	Yes (N, %)	16 (32 %)	0 (0 %)
	No (N, %)	23 (46 %)	6 (60 %)
	Unknown (N, %)	11 (22 %)	4 (40 %)
Pre-RT dental extractions			
	Yes (N, %)	17 (34 %)	3 (30 %)
	No (N, %)	33 (66 %)	7 (70 %)
Number of missing teeth (mean, range)		6 (0–18)	6 (1–9)

* Oral hygiene status was defined by our Oral Oncology providers based on clinical and radiographic imaging findings such as the presence of plaque, calculus (tartar build-up) and gingiva inflammation (gingivitis).

### 3.2 Model performance

**Fig. 2:**
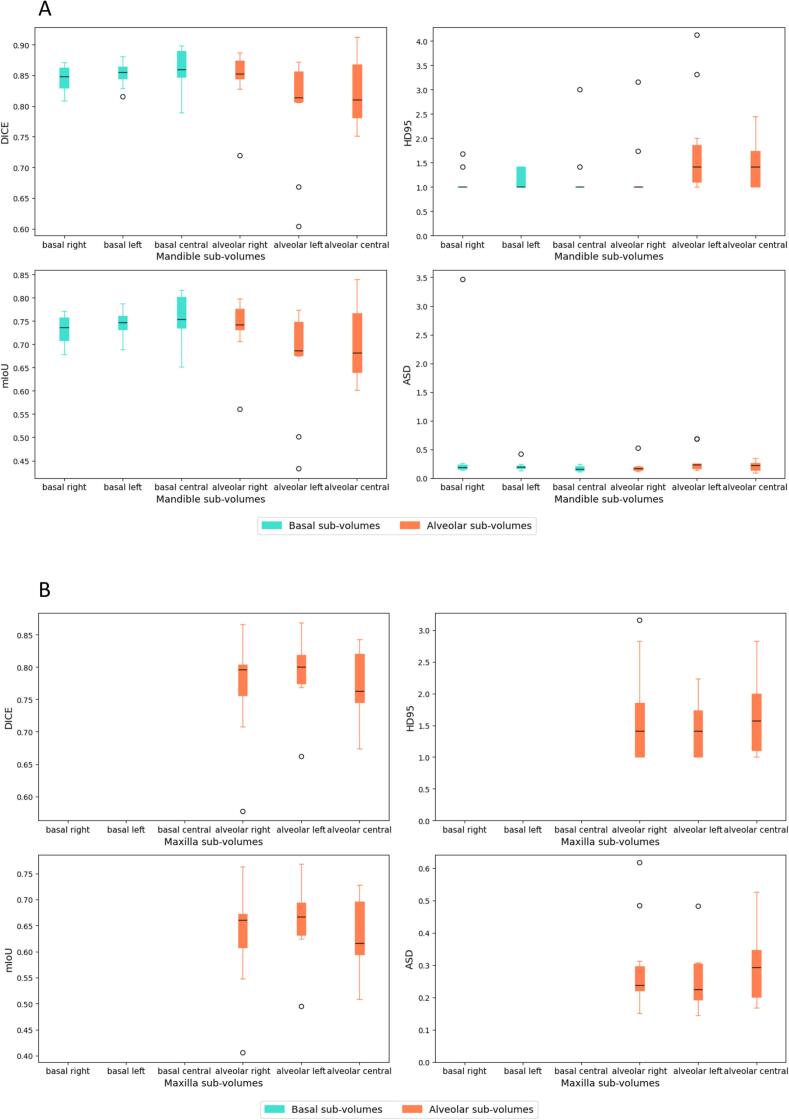

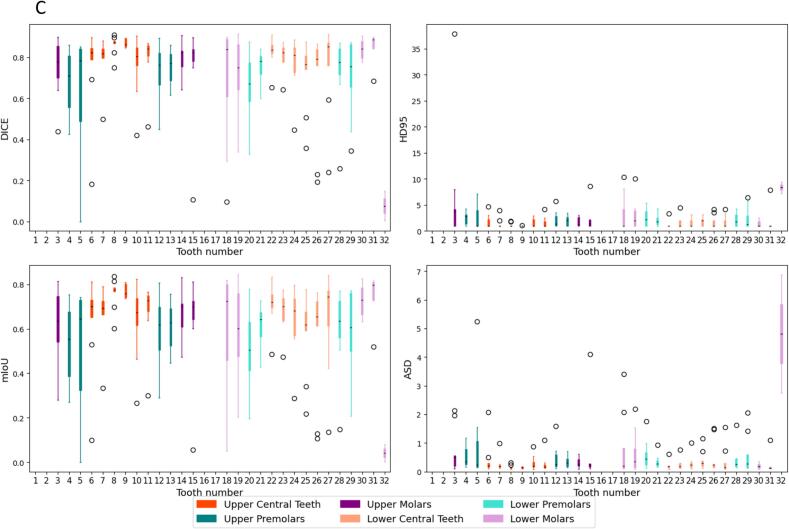
Segmentation performance. Boxplots describing the distribution of performance metrics by class for the A) mandible sub-volumes B) maxilla sub-volumes and C) teeth segmentation models.

The training and validation subset consisted of 50 patients and 10 randomly selected cases were used for testing. Successful segmentation of mandible sub-volumes was achieved, with basal sub-volumes showing overall higher segmentation accuracy compared to alveolar sub-volumes (Fig. 2a). In comparison to the mandible, the segmentation performance for maxilla sub-volumes showed notable differences (Fig. 2b). Maxilla basal sub-volumes were not successfully segmented, most likely due to limited training data for this often small and even non-existing sub-volume. However, both maxilla and mandible alveolar sub-volumes exhibited low average surface distances, indicating consistent precision in boundary delineation. Full quantitative metrics are provided in Table 2.

**Table 2:** Segmentation performance. Mandible and maxilla sub-volumes and teeth segmentation models’ segmentation performance results. Mandible and maxilla sub-volumes were grouped by the following regions: basal (classes 1–3) vs. alveolar (classes 4–6). Teeth were grouped as follows: Upper Central (6–11), Upper Premolars (4, 5, 12, 13), Upper Molars (1–3, 14–16), Lower Central (22–27), Lower Premolars (20, 21, 28, 29) and Lower Molars (17–19, 30–32).

Anatomical region	Dice Mean (range)	mIoU Mean (range)	HD (mm) Mean (range)	ASD (mm) Mean (range)
Mandible basal	0.85 (0.80–0.88)	0.74 (0.67–0.79)	1.2 (1.0–2.0)	0.3 (0.1–1.4)
Mandible alveolar	0.82 (0.69–0.89)	0.70 (0.53–0.80)	1.5 (1.0–3.2)	0.2 (0.1–0.5)
Maxilla alveolar	0.78 (0.64–0.86)	0.64 (0.47–0.75)	1.6 (1.0–2.7)	0.3 (0.2–0.5)
Upper central teeth	0.80 (0.53–0.89)	0.69 (0.39–0.81)	1.4 (1.0–3.1)	0.3 (0.1–0.9)
Upper premolars	0.69 (0.37–0.87)	0.56 (0.25–0.76)	2.3 (1.0–5.1)	0.6 (0.1–2.2)
Upper molars	0.76 (0.40–0.90)	0.64 (0.27–0.82)	3.7 (1.0–16.5)	0.6 (0.1–2.3)
Lower central teeth	0.76 (0.42–0.89)	0.63 (0.28–0.80)	1.6 (1.0–3.7)	0.3 (0.1–1.1)
Lower premolars	0.71 (0.38–0.86)	0.57 (0.25–0.76)	2.3 (1.0–5.1)	0.5 (0.1–1.6)
Lower molars	0.63 (0.38–0.75)	0.54 (0.28–0.68)	3.6 (2.3–8.0)	1.4 (0.6–2.8)

The teeth segmentation model demonstrated variable performance across different tooth groups (Fig. 2c), which can be partially attributed to the proportion of missing teeth in the datasets (see Figs. S5 and S6 in Supplement D). Central teeth, both upper and lower, which achieved higher segmentation accuracy, also had lower percentages of missing data in both the training and test datasets. In contrast, premolars and molars, particularly upper molars, exhibited higher percentages of missing data, correlating with their lower Dice scores and mIoU values. Central teeth, both upper and lower, achieved higher segmentation accuracy compared to premolars and molars (Fig. 2c).

### 3.3 Dose-volume comparison

**Fig. 3:**
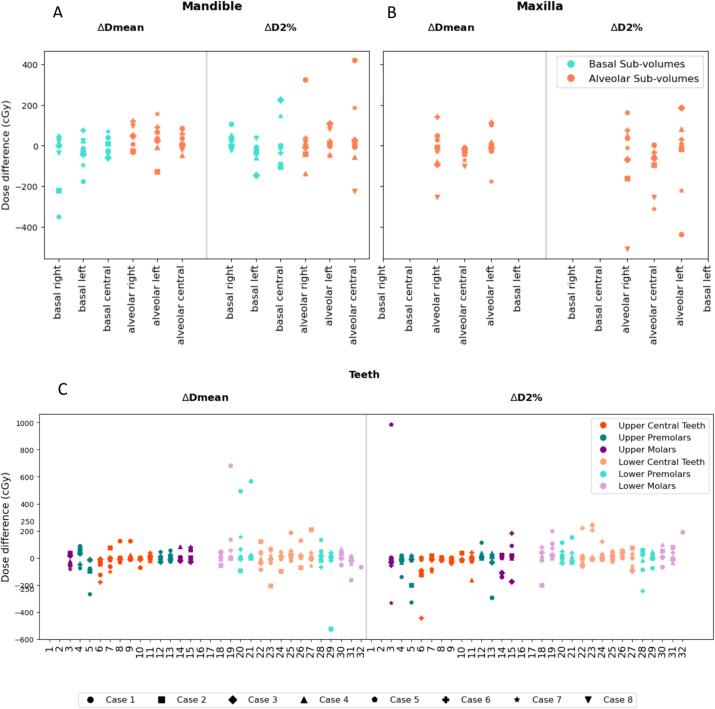
Dose-volume comparison. Differences in dose-volume metrics (ΔD_2%_ and ΔD_mean_) between predicted and ground truth orodental structures – mandible sub-volumes (A), maxilla sub-volumes (B) and teeth (C) – for 8 test cases.

Fig. 3 shows a dose-volume comparison between predicted class segmentations and manually contoured structures for 8 cases (two of the test cases had radiation dose fields far from the jaws with no dose delivered to the structures of interest). While most cases fell within the ± 2.5 Gy dose difference range for D_mean_ in the mandible and maxilla sub-volumes, more outliers were observed for D_2%_. The dose-volume difference observed for the maxilla alveolar central sub-volume was found to be statistically significant for both D_mean_ (adjusted p-value = 0.01) and D_2%_ (adjusted p-value = 0.04).

## 4 Discussion

This work presents a novel approach to the auto-segmentation of orodental structures that has the potential of supporting clinical decision-making in radiation therapy for patients with head and neck cancer.

Osteoradionecrosis of the mandible and maxilla remains a significant late complication of head and neck radiotherapy, and its clinical management is challenged by limitations in early detection and severity stratification. The recently ASCO-endorsed ClinRad ORN staging system [16] was developed to improve diagnostic consistency and guide intervention strategies by standardizing radiographic classification of ORN lesions according to anatomical location and vertical extent of bone involvement (i.e., distinguishing between alveolar and basal bone damage). Despite this recent progress, several important gaps in clinical practice still limit effective ORN risk assessment, including the absence of standardized and reproducible delineation of orodental structures, insufficient understanding of how radiation dose to specific sub-volumes correlates with ORN development, and unclear contribution of pre-existing dental pathology such as periodontal disease or caries to subsequent ORN risk. Advancing beyond existing orodental auto-segmentation methods [[5], [6], [7], [8]], our study proposes a DL framework that enables anatomically granular and reproducible auto-segmentation of orodental sub-volumes that explicitly align with the ClinRad system and provides a critical step toward addressing these gaps by supporting the development of spatially informed NTCP models and image-based ORN detection tools.

The clinical applicability of our models expands to a wider range of clinical settings related but not exclusive to ORN staging. Image-based features from computed tomography (CT) images have been associated to ORN regions [23,24]. We have previously demonstrated that ORN [25] or even earlier stages of mandible damage (e.g., microvascular damage) [26] can be detected on magnetic resonance images (MRI); ongoing work is focused on black bone MRI for ORN detection [27]. However, a recent survey [28] has shown the poor performance of clinician-based radiographic diagnosis of mandibular damage leading to high variability in the application of the ClinRad ORN staging system, thus establishing a benchmark for more advanced computerized alternatives to the detection of pathological bony changes. Automated sub-volume-based assessments of pre-existing oral conditions can support image-based early detection of bony changes resulting in more timely and granular interventions for reduced risk of more severe radiation-induced complications such as ORN. The proposed sub-volume definitions also offer a promising framework for incorporating anatomical specificity into Normal Tissue Complication Probability (NTCP) modelling to support the identification of high-risk regions. Sub-volume-based NTCP models could allow for more accurate dose-toxicity correlations by capturing spatial heterogeneity in bone composition and radiosensitivity. On the other hand, our proposed sub-volumes could allow for more detailed dosimetric optimization during treatment planning, with potential adaptation of radiotherapy plans to reduce the risk of radiation-induced orodental complications before they occur.

Our study had some limitations. Although the models achieved high overall segmentation performance, which directly translated into dosimetric accuracy, they exhibited limited applicability in segmenting teeth and sub-volumes often absent in the data (e.g., molars, premolars, maxilla alveolar sub-volumes). Missing data both in the training and test datasets may influence the performance of the models (Supplementary Fig. S6). Underrepresented structures in the training dataset clearly affected model performance, as the model cannot learn the features of the missing teeth or sub-volumes. On the other hand, underrepresentation of structures in the test dataset may affect reliability of the performance measures, as the computed metrics are based on limited data.

Another potential limitation of our study is its applicability to images with significant artifacts. Although we excluded cases with overt artifacts during model training, minimal artifacts could still have adversely affected model performance at inference time. A further consideration relates to the heterogeneity of the training dataset, which included scans acquired across multiple scanner manufacturers and acquisition protocols (see Supplement C). Moreover, each CT scanner applies its own proprietary metal artifact reduction algorithm during image reconstruction, which can introduce variability in image appearance. While such variation may introduce a degree of noise into the training data, it may also improve model robustness by exposing it to real-world imaging variability. Nevertheless, it is worth noting that 50 out of the 60 cases in our training dataset were acquired using Philips scanners, providing a relatively consistent imaging.

In parallel to the proposed DL-based auto-segmentation approach, our group is currently working on a semi-automated teeth segmentation approach, the RADiation dose MAPping tool (RADMAP) tool, using an angular ray-based algorithm which is agnostic to missing teeth. While this tool is not fully automated and requires some manual adjustments on the angular rays defining the boundaries between teeth, it allows for domain knowledge input with regards to missing and shifted teeth or with artifact-heavy images. Future work will explore using the RADMAP approach to inform our DL-based teeth auto-segmentation model for a more generalized applicability.

Finally, establishing the boundaries between sub-volumes in the mandible and maxilla is challenging. The extent of the alveolar process varies across the mandible and maxilla structures [29] and between patients [30] and can be affected by dental extractions [14]. After dental extractions the size of the alveolar ridge decreases as it is resorbed and evolves into a so-called ‘residual ridge’ which is formed by denser cortical and trabecular bone with up to a 50 % reduction in the height of the alveolar socket [14,31]. This process is typically more obvious in the molar and premolar areas, where teeth extractions are more common. For this study, a standardized alveolar process height of 3 to 5 mm was applied across all cases to define the alveolar sub-volumes of the maxilla and mandible. However, these dimensions represent a generalized approach and do not fully account for the variability inherent in individual anatomy and dental factors, which can significantly influence the structure and dimensions of the alveolar region, particularly in older edentulous patients. Future work will focus on refining our methodology to further advance towards a patient-specific approach to sub-volume definition and modeling.

In conclusion, this study presents a novel framework for DL-based auto-segmentation of orodental structures to develop a radiation-specific tool capable of spatially localizing dose-related differences in the jaw, thereby supporting a more effective approach to image-based bone damage detection, including ORN, and improving clinical decision-making in radiation oncology and dental care for head and neck cancer patients.

## Declaration of Generative AI and AI-assisted technologies in the writing process

During the preparation of this work, the authors used ChatGPT (GPT-4 architecture) to improve the grammatical accuracy and semantic structure of portions of the text. After using this tool, the authors reviewed and edited the content as needed and take full responsibility for the content of the publication

## CRediT authorship contribution statement

**Laia Humbert-Vidan:** Conceptualization, Methodology, Software, Validation, Formal analysis, Investigation, Data curation, Writing – original draft, Writing – review & editing, Visualization. **Austin H. Castelo:** Conceptualization, Methodology, Software, Validation, Formal analysis, Investigation, Data curation, Writing – original draft, Writing – review & editing. **Renjie He:** Conceptualization, Methodology, Software, Validation, Formal analysis, Investigation, Data curation, Writing – original draft, Writing – review & editing. **Lisanne V. van Dijk:** Methodology, Writing – review & editing. **Dong Joo Rhee:** Investigation, Resources, Data curation, Writing – review & editing. **Congjun Wang:** Investigation, Writing – review & editing. **He C. Wang:** Investigation, Resources, Writing – review & editing. **Kareem A. Wahid:** Methodology, Writing – review & editing. **Sonali Joshi:** Data curation, Writing – review & editing. **Parshan Gerafian:** Data curation, Writing – review & editing. **Natalie West:** Data curation, Writing – review & editing. **Zaphanlene Kaffey:** Data curation, Writing – review & editing. **Sarah Mirbahaeddin:** Data curation, Writing – review & editing. **Jaqueline Curiel:** Data curation, Writing – review & editing. **Samrina Acharya:** Data curation, Writing – review & editing. **Amal Shekha:** Data curation, Writing – review & editing. **Praise Oderinde:** Data curation, Writing – review & editing. **Alaa M.S. Ali:** Data curation, Writing – review & editing. **Andrew Hope:** Writing – review & editing. **Erin Watson:** Writing – review & editing. **Ruth Wesson-Aponte:** Writing – review & editing. **Steven J. Frank:** Writing – review & editing. **Carly E.A. Barbon:** Writing – review & editing. **Kristy K. Brock:** Resources, Writing – review & editing. **Mark S. Chambers:** Resources, Writing – review & editing. **Muhammad Walji:** Writing – review & editing. **Katherine A. Hutcheson:** Writing – review & editing. **Stephen Y. Lai:** Resources, Writing – review & editing. **Clifton D. Fuller:** Conceptualization, Supervision, Software, Validation, Formal analysis, Investigation, Resources, Writing – original draft, Writing – review & editing. **Mohamed A. Naser:** Conceptualization, Methodology, Software, Validation, Formal analysis, Investigation, Resources, Data curation, Writing – original draft, Writing – review & editing. **Amy C. Moreno:** Conceptualization, Methodology, Supervision, Project administration, Software, Validation, Formal analysis, Investigation, Resources, Data curation, Writing – original draft, Writing – review & editing.

## Declaration of competing interest

The authors declare the following financial interests/personal relationships which may be considered as potential competing interests: CDF has received travel, speaker honoraria, and/or registration fee waivers unrelated to this project from Siemens Healthineers/Varian, Elekta AB, Philips Medical Systems, The American Association for Physicists in Medicine, The American Society for Clinical Oncology, The Royal Australian and New Zealand College of Radiologists, Australian & New Zealand Head and Neck Society, The American Society for Radiation Oncology, The Radiological Society of North America, and The European Society for Radiation Oncology. KAW serves as an Editorial Board Member for Physics and Imaging in Radiation Oncology. The authors declare that no other competing interests exist.
