# Supplementary material for: Image-based mandibular and maxillary parcellation and annotation using computed tomography (IMPACT): a deep learning-based clinical tool for orodental dose estimation and osteoradionecrosis assessment
Source: Phys Imaging Radiat Oncol. 2025 Jul 25;35:100817. doi: 10.1016/j.phro.2025.100817 (PMC12398235; doi:10.1016/j.phro.2025.100817)
Supplement: Supplementary Data 1 [file mmc1.pdf]

Supplement A. Extracts from the contouring instructions provided to observers

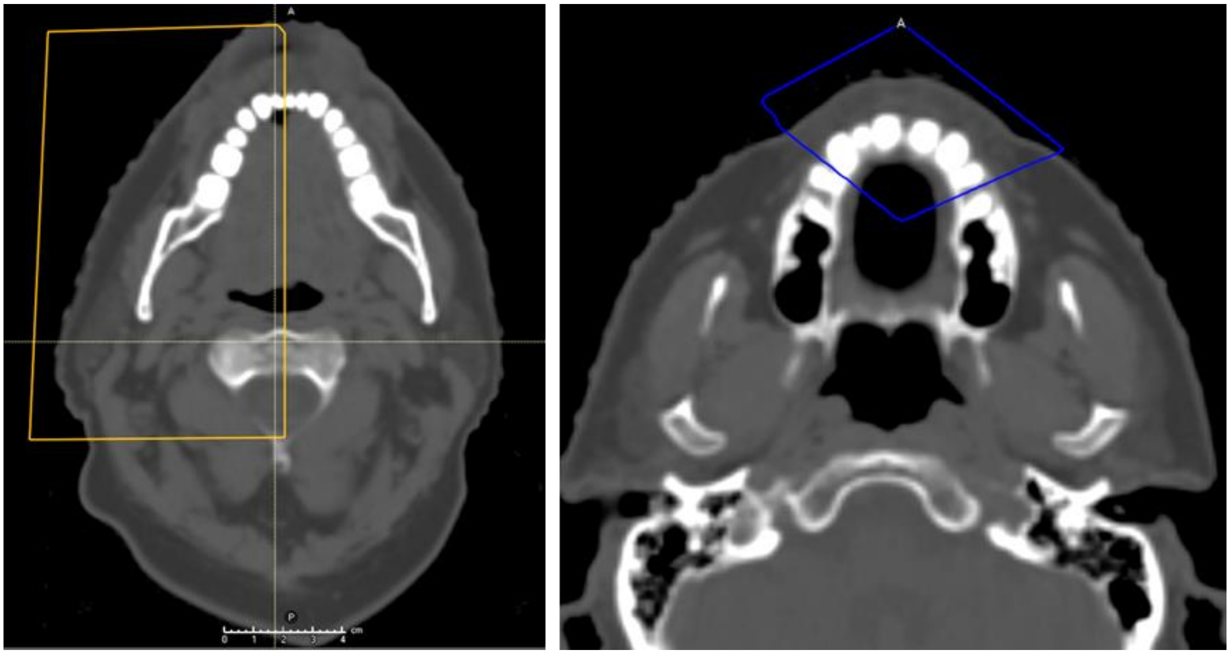

Figure S1. Determination of laterality and central section. The RayStation contouring tool ‘Polygon’ was used to create ‘boxes’ to assist with defining the laterality (left/central/right) in the mandible and maxilla. A first rectangular box (left image) was used to determine left/right using the central incisors to guide the positioning of the midline. A second box (right image) was used to determine the central sections, with the limits determined by the space between the canines and first premolars.

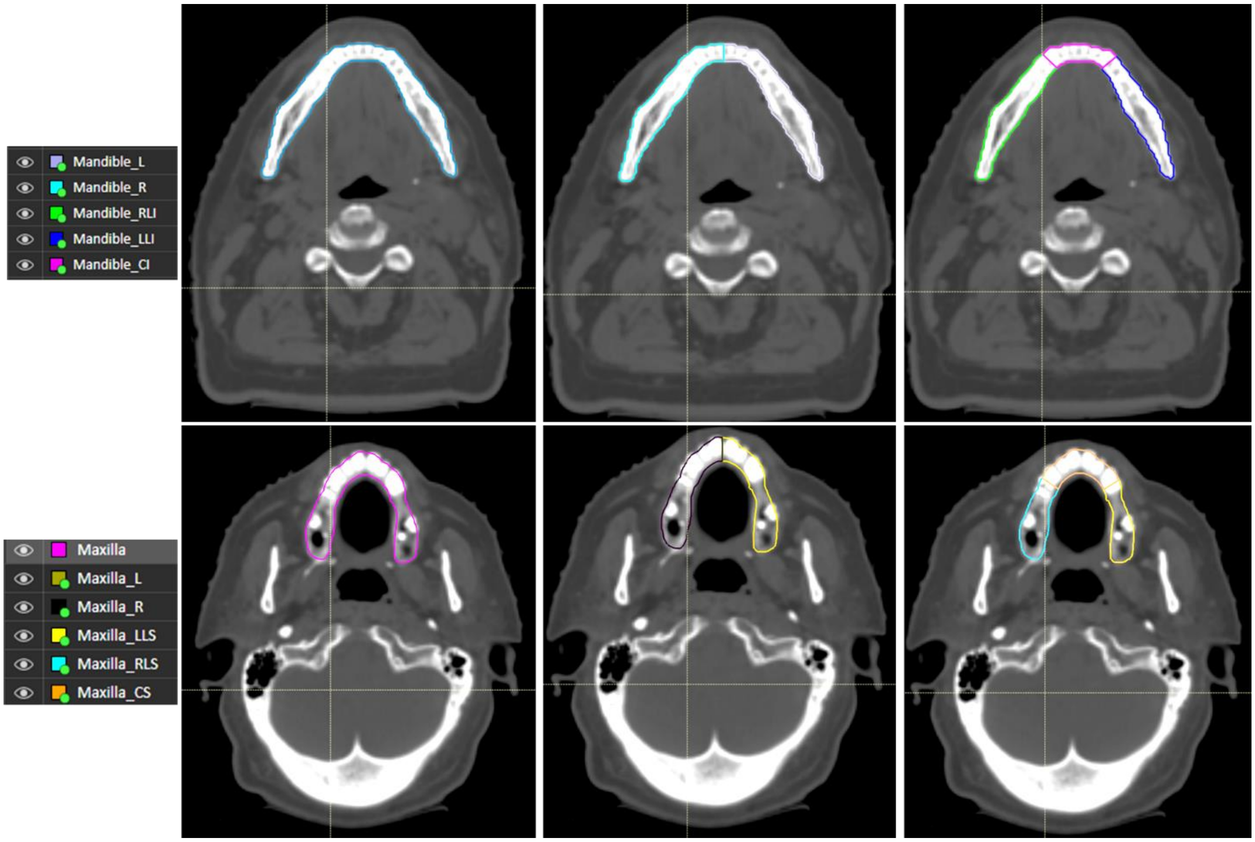

Figure S2. Mandible and maxilla sub-volumes overview. Axial slices demonstrating the whole mandible/maxilla contours (left), laterality separation (middle) and final sub-volumes (right). Specific algebra instructions were provided within the RayStation contouring template to achieve these contours, where 'UpperR'/'LowerR' correspond to the maxilla/mandible laterality rectangle boxes and 'UpperBox'/'LowerBox' correspond to the maxilla/mandible central section boxes: Maxilla_R = Maxilla + Intersection + UpperR; Maxilla_L = Maxilla + Substraction + UpperR; Maxilla_LLS = Maxilla + Substraction + UpperR & UpperBox; Maxilla_RLS = Maxilla + Substraction + UpperBox & Maxilla_LLS; Maxilla_CS = Maxilla + Substraction + Maxilla_RLS & Maxilla_LLS; Mandible_R = Bone_Mandible + Intersection + LowerR; Mandible_L = Bone_Mandible + Substraction + LowerR; Mandible_LLI = Bone_Mandible + Substraction + LowerR & LowerBox; Mandible_RLI = Mandible + Substraction + LowerBox & Mandible_LLI; Mandible_CI = Bone_Mandible + Substraction + Mandible_RLI & Mandible_LLI

Supplement B. Mandible and maxilla sub-volumes contouring

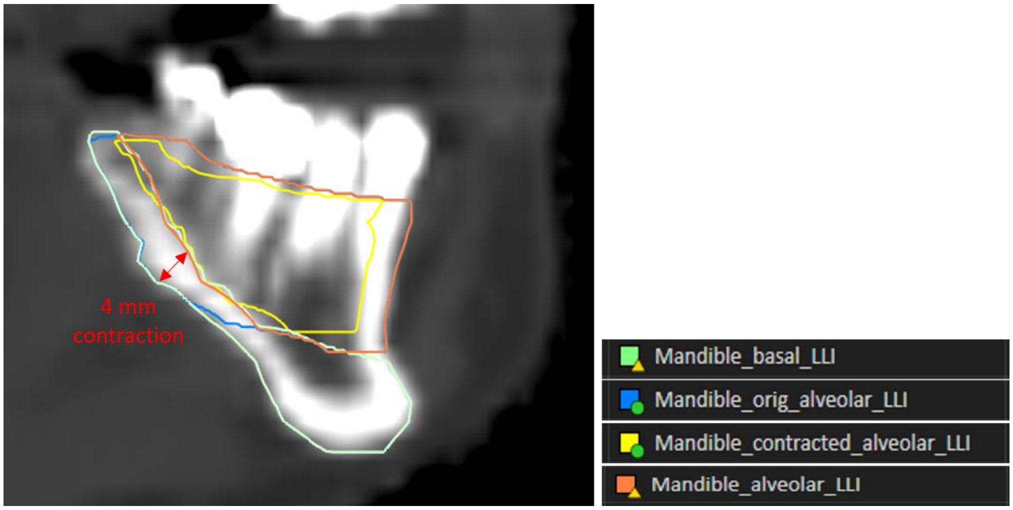

Figure S3. Contouring example. Example demonstrating the need for an adjustment of the alveolar sub-volumes at the level of the molars in order to allow for a differentiation between alveolar and basal regions. For this, the standard 5 mm expansion corresponding to the alveolar region was adjusted by 1 mm using a 4 mm 'contraction' of the mandible contour at the molars level.

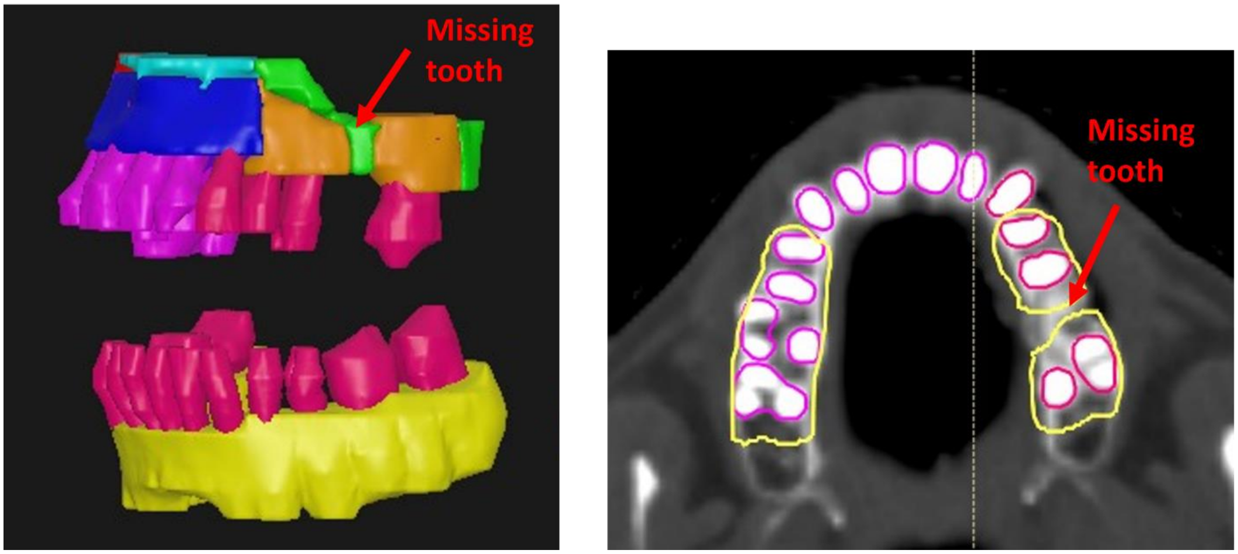

Figure S4. Contouring example. Example of a case with a missing tooth. To ensure generalizability of the model on edentulous or semi-edentulous patients, empty tooth sockets in cases with missing teeth were included in the alveolar region by manually adjusting the teeth expansion contour.

Supplement C. Overview of technical details on CT scans

Technical detail	Values	N	%
Exposure (mAs)	175	17	28.30%
	300	7	11.70%
	250	4	6.70%
	500	4	6.70%
	375	3	5.00%
	489	3	5.00%
	350	3	5.00%
	420	3	5.00%
	450	2	3.30%
	37	2	3.30%
	405	2	3.30%
	400	2	3.30%
	385	1	1.70%
	410	1	1.70%
	376	1	1.70%
	73	1	1.70%
	425	1	1.70%
	221	1	1.70%
	46	1	1.70%
	27	1	1.70%
Energy (kVp)	120	54	90.00%
	140	5	8.30%
	100	1	1.70%
Manufacturer	Philips	50	83.30%
	GE Medical Systems	5	8.30%
	Siemens	4	6.70%
	Unknown	1	1.70%
Pixel Spacing (mm)	[0.9765625, 0.9765625]	40	66.67%
	[1.171875, 1.171875]	11	18.30%
	[1.03515625, 1.03515625]	6	10.00%
	[1.36523438, 1.36523438]	2	3.30%
	[1.35742188, 1.35742188]	1	1.70%
Reconstruction Diameter (mm)	500	39	65.00%
	600	11	18.30%
	530	7	11.70%
	699	2	3.30%
	695	1	1.70%
Slice Thickness (mm)	3	32	53.30%
	2.5	23	38.30%
	2	3	5.00%
	1	1	1.70%
	3.75	1	1.70%

Supplement D. Missing teeth analyses

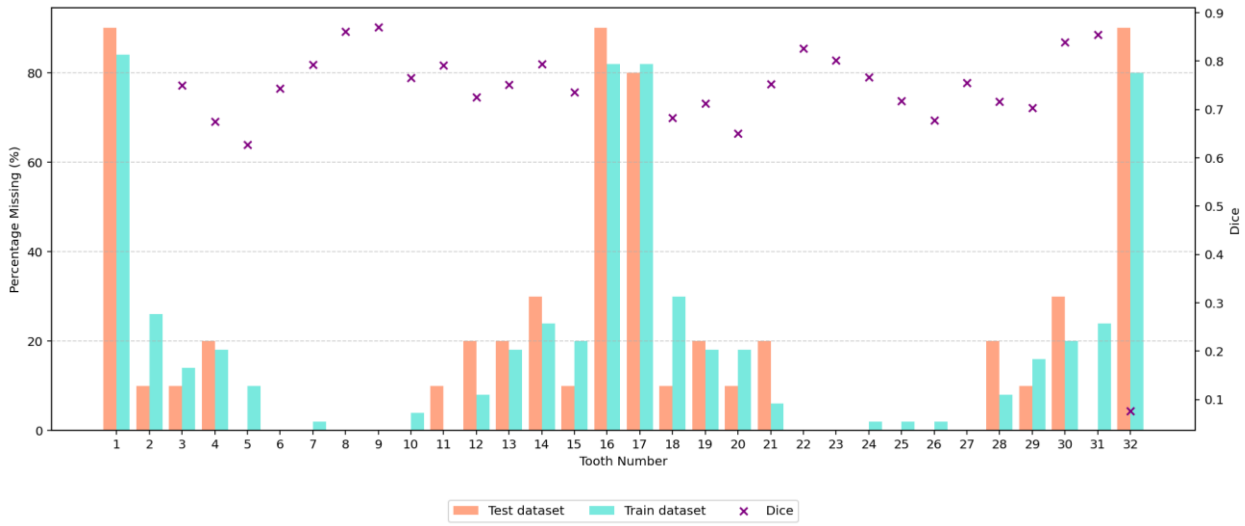

Figure S5. Missing teeth percentage distribution. Bar chart illustrating the distribution of missing teeth percentages across the training and test datasets, with corresponding Dice metric values overlaid as purple markers. Teeth with higher missing percentages, such as the first and last molars (teeth 1, 16, 17, 32), exhibit lower Dice scores, indicating poorer segmentation performance in these regions. Conversely, teeth with lower missing percentages tend to have higher Dice scores, reflecting better segmentation accuracy.

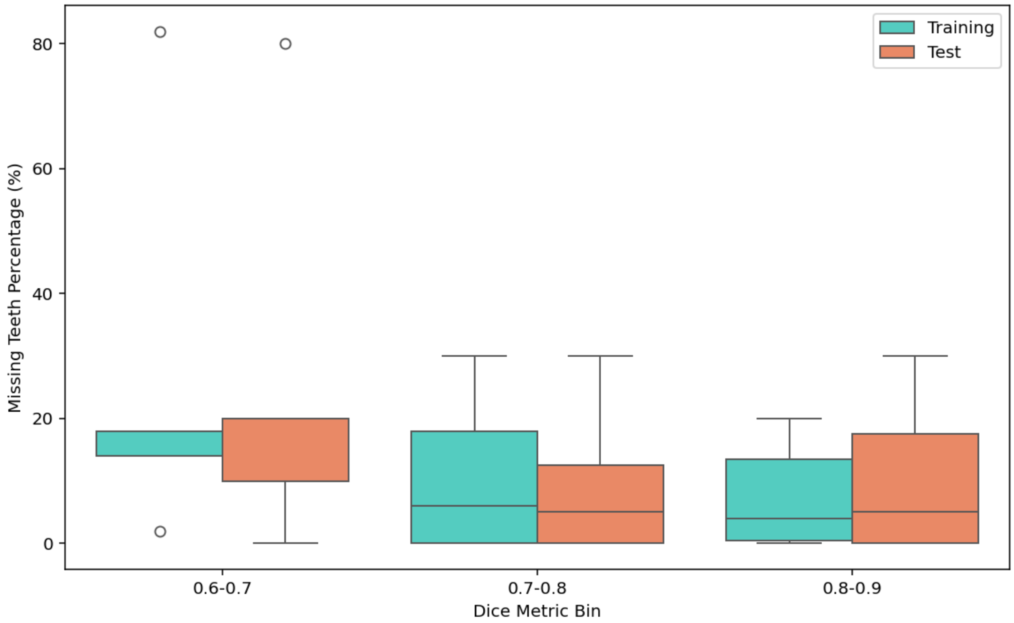

Figure S6. Missing teeth percentage distribution. Boxplot showing the distribution of missing teeth percentages in training and test datasets, grouped by Dice metric bins. The results indicate an inverse relationship between Dice similarity and the percentage of missing teeth, with lower Dice scores (0.6–0.7) associated with higher missing teeth percentages. As Dice scores improve (≥0.7), the median and spread of missing teeth percentages decrease, suggesting better segmentation performance in cases with fewer missing teeth.

Supplement E. Checklist for Artificial Intelligence in Medical Imaging (CLAIM)

Section / Topic	No.	Item	Page / Line	No	NA
TITLE / ABSTRACT					
	**1**	Identification as a study of AI methodology, specifying the category of technology used (e.g., deep learning)	**1**		
ABSTRACT					
	**2**	Summary of study design, methods, results, and conclusions	**2**		
INTRODUCTION					
	**3**	Scientific and/or clinical background, including the intended use and role of the AI approach	**3**		
	**4**	Study aims, objectives, and hypotheses	**3**		
METHODS					
Study Design	**5**	Prospective or retrospective study	**4**		
	**6**	Study goal	**4**		
Data	**7**	Data sources	**4**		
	**8**	Inclusion and exclusion criteria	**4**		
	**9**	Data pre-processing	**5**		
	**10**	Selection of data subsets	**5/6**		
	**11**	De-identification methods	**n/a**		
	**12**	How missing data were handled	**5/6**		
	**13**	Image acquisition protocol	**Supplement C**		
Reference Standard	**14**	Definition of method(s) used to obtain reference standard	**4**		
	**15**	Rationale for choosing the reference standard	**4**		
	**16**	Source of reference standard annotations	**4**		
	**17**	Annotation of test set	**4**		
	**18**	Measures of inter- and intra-rater variability of features described by the annotators	**n/a**		**x**
Data Partitions	**19**	How data were assigned to partitions	**6**		
	**20**	Level at which partitions are disjoint	**6**		
Testing Data	**21**	Intended sample size	**6**		

Section / Topic	No.	Item	Page / Line	No	NA
Model	**22**	Detailed description of model	**5/6**		
	**23**	Software libraries, frameworks, and packages	**5/6**		
	**24**	Initialization of model parameters	**n/a**		
Training	**25**	Details of training approach	**5/6**		
	**26**	Method of selecting the final model	**5/6**		
	**27**	Ensembling techniques	**5/6**		
Evaluation	**28**	Metrics of model performance	**6**		
	**29**	Statistical measures of significance and uncertainty	**6**		
	**30**	Robustness or sensitivity analysis	**n/a**		**x**
	**31**	Methods for explainability or interpretability	**n/a**		**x**
	**32**	Evaluation on internal data	**n/a**		**x**
	**33**	Testing on external data	**6**		
	**34**	Clinical trial registration	**n/a**		**x**
RESULTS					
Data	**35**	Numbers of patients or examinations included and excluded	**6/7**		
	**36**	Demographic and clinical characteristics of cases in each partition	**6/7**		
Model performance	**37**	Performance metrics and measures of statistical uncertainty	**7/8**		
	**38**	Estimates of diagnostic performance and their precision	**10/11**		
	**39**	Failure analysis of incorrect results	**n/a**		**x**
DISCUSSION					
	**40**	Study limitations	**11/12**		
	**41**	Implications for practice, including intended use and/or clinical role	**11/12**		
OTHER INFORMATION					
	**42**	Provide a reference to the full study protocol or to additional technical details	**n/a**		**x**
	**43**	Statement about the availability of software, trained model, and/or data	**1**		
	**44**	Sources of funding and other support; role of funders	**13**		
